# PrEP Care Continuum Engagement Among Persons Who Inject Drugs: Rural and Urban Differences in Stigma and Social Infrastructure

**DOI:** 10.1007/s10461-021-03488-2

**Published:** 2021-10-09

**Authors:** Suzan M. Walters, David Frank, Brent Van Ham, Jessica Jaiswal, Brandon Muncan, Valerie Earnshaw, John Schneider, Samuel R. Friedman, Danielle C. Ompad

**Affiliations:** 1 Department of Epidemiology, New York University School of Global Public Health, New York, NY USA; 2 Center for Drug Use and HIV/HCV Research, New York, NY USA; 3 Department of Population Science and Policy, SIU School of Medicine, Carbondale, IL USA; 4 Department of Health Science, University of Alabama, Tuscaloosa, AL USA; 5 Renaissance School of Medicine at Stony, Brook University, Stony Brook, NY USA; 6 Department of Human Development and Family Sciences, University of Delaware, Newark, DE USA; 7 Department of Medicine, University of Chicago, Chicago, IL USA; 8 Department of Population Health, New York University Grossman School of Medicine, New York, USA

**Keywords:** Pre-exposure prophylaxis (PrEP), Persons who inject drugs (PWID), Stigma, HIV, Social infrastructure, Third places, Rural, Urban

## Abstract

Pre-exposure prophylaxis (PrEP) is a medication that prevents HIV acquisition, yet PrEP uptake has been low among people who inject drugs. Stigma has been identified as a fundamental driver of population health and may be a significant barrier to PrEP care engagement among PWID. However, there has been limited research on how stigma operates in rural and urban settings in relation to PrEP. Using in-depth semi-structured qualitative interviews (n = 57) we explore PrEP continuum engagement among people actively injecting drugs in rural and urban settings. Urban participants had more awareness and knowledge. Willingness to use PrEP was similar in both settings. However, no participant was currently using PrEP. Stigmas against drug use, HIV, and sexualities were identified as barriers to PrEP uptake, particularly in the rural setting. Syringe service programs in the urban setting were highlighted as a welcoming space where PWID could socialize and therefore mitigate stigma and foster information sharing.

**Supplementary Information:**

The online version contains supplementary material available at 10.1007/s10461-021-03488-2.

## Introduction

People who inject drugs (PWID) can benefit from pre-exposure prophylaxis (PrEP) to prevent HIV [1]. The Bangkok Tenofovir Study, which has been the only randomized trial among PWID, found PrEP to be 74%-84% effective among PWID when taken regularly [1, 2]. Studies among sexual minority men and transgender women, which focus on sexual transmission, have found about 99% efficacy when taken regularly [3, 4]. Despite the benefit of PrEP, uptake among PWID has been low, with recent studies reporting between 1 and 2% [5, 6]. One barrier to PrEP uptake is low PrEP awareness [5, 7, 8]. Yet, when informed about PrEP, many PWID are interested in taking it. For example, one study reported 63% willingness to take PrEP among PWID in Baltimore, Maryland [5], and another study found 59% among PWID in San Francisco and Los Angeles, California [6]. In addition to awareness, barriers to uptake include co-pays for doctors’ visits, the need to take PrEP daily, and concerns about increased risk for HIV or sexually transmitted infections with PrEP [6]. In addition, PrEP prescribers are less willing to prescribe PrEP to PWID, compared to sexual minority men who do not inject drugs [9, 10]. The lack of willingness to prescribe to PWID may, in part, be due to drug use and other stigmas held by providers [11, 12], which may be exacerbated in rural settings [13].

In 2019, injection drug use accounted for 2.3% of new HIV diagnoses in New York City, and men who have sex with men who also inject drugs accounted for 1.5% [14]. In Illinois, during 2013–2017, 2.1% of HIV incidence were attributed to injection drug use, and an additional 2.5% to men who have sex with men who also inject drugs [15]. Importantly, there have been several recent HIV outbreaks among PWID in the United States (US) attributed to injection drug use [16–19]. Although rural populations in the US have been disproportionately burdened by the opioid crisis [20–25], in 2019 New York City (NYC) issued an advisory that risk factors that contribute to HIV outbreaks were prevalent among PWID [26]. As such, PrEP could be useful in both areas. Most studies examining PrEP have focused on urban populations leaving a gap in our understanding about the lives of rural PWID, and how their HIV risk and engagement with PrEP may differ from urban PWID [27]. For example, rural PWID likely have fewer resources for healthcare and harm reduction [27] and experience greater stigma due to drug use [28], all of which could increase HIV risk. Furthermore, late HIV diagnoses happen more frequently in rural areas, compared to urban areas [29] and rural areas have lower rates of retention in care and viral suppression [30]. However, little is known about differences among rural and urban populations regarding PrEP care continuum engagement nor about preferred ways to take PrEP.

This study sought identify barriers to and facilitators of PrEP use among rural and urban PWID. We use the PrEP care continuum [31, 32], which outlines the sequential steps of PrEP care [33], as a framework to identify specific areas for PrEP care that PWID are engaged or disengaged with, focusing on four stages: awareness, knowledge, willingness, and uptake. We emphasize PrEP knowledge because research with PWID, although limited, has suggested that this population has PrEP awareness (i.e., they have heard of it), but lack a full understanding of what PrEP is and does (i.e., they do not know what purpose it serves, what it protects against, or how it is used) [34]. This gap may be especially pronounced in rural settings, as a recent study in a rural community found that 68% of PWID (n = 48) reported PrEP awareness but none were able to accurately describe it [27].

## Theoretical Framework

### Stigma as a Fundamental Cause

HIV is socially constructed [35, 36], socially transmitted [36, 37], and socially located [36, 38]. HIV spreads through groups of people and social networks, and therefore, HIV is “fundamentally a social phenomenon” [39]. Rather than focusing on individual behaviors as risks for HIV, fundamental cause theory centers disease burden around the social conditions that place people “at risk for risks” [40]. Fundamental cause theory posits that social conditions are central to patterns of disease since they determine a person’s ability to manage their health. Fundamental causes shape access to resources, including those that would promote PrEP awareness and knowledge, that help prevent disease. They influence multiple disease outcomes (e.g., overdose, HIV, and HCV) through multiple risk factors and are linked to poorer health outcomes over time.

Importantly, when medical interventions (e.g., PrEP) are developed disparities widen [40, 41]. For example, Black and Hispanic sexual minority men (SMM) have lower PrEP uptake than white SMM, despite having higher HIV prevalence [42]. Similarly Black and Hispanic women, have lower PrEP uptake and higher HIV prevalence, compared to white women [43]. The social processes involving resources, power, capital etc. are allowing for increased life expectancy among higher income and white populations.

This study focuses on stigma due to drug use, sexualities, and HIV as a fundamental cause of PrEP disparities. Drug use stigma permeates US society in policies criminalizing drug use and administering drug treatment as well as through public stigma in stereotypes and actions toward people using drugs [44]. Once people who use drugs encounter discriminatory treatment, they begin to anticipate discrimination in the future and in some cases, they begin to internalize stigmatizing beliefs about themselves [45–49]. All of this negatively impacts mental and physical health [50–52]. Although drug use stigma intersects with other forms of stigma, which create unique situations as we will describe in relation to PrEP, drug use stigma powerfully shapes the lives of PWID. This may be because drug use stigma can be more intense and lasts longer compared to other forms of stigma, such as stigma associated with smoking or obesity [53]. Moreover, there is a hierarchy by drug type, creating gradations of stigma, with injection drug use being the most stigmatized particularly in regard to HIV risk [50, 54]. Thus, the very fact that someone injects drugs may overpower any other characteristic they hold, creating a master status, and consequently being treated as though they have a “spoiled identity” [55, 56]. Recent research has begun to grapple with how such stigmatized identities can be fundamental causes to health disparities [57–60]. However, research has mainly focused on stigma due to sexual orientation [57–60]. We add to the literature by focusing on drug use stigma experienced by PWID and how sexualities and HIV stigmas intersect to create barriers to PrEP. Specifically, we demonstrate how stigma related to drug use often halted care continuum engagement at the first two steps, awareness and knowledge.

### Third Places and Social Infrastructure

Syringe service programs (SSPs) are often stigma-free spaces that promote health collectivity [61] by providing a physical space for social networks to form [62–65]. SSPs can be fixed-locations where people go for services or they can be mobile where goods and services are delivered directly to clients in their communities. SSPs not only supply new injecting equipment, but many dispose of used syringes, conduct HIV and HCV testing, provide condoms and other safer sex items, and provide referrals to other resources [66] including housing services [67], and some SSPs offer on-site healthcare services such as HCV care [68] and drug treatment [69]. For all of these reasons, SSPs are associated with reduced HIV transmission [70]. Importantly, but less talked about, is the benefits of a physical space where people can congregate for social purposes, which can facilitate building social ties and networks and improve health outcomes [71, 72]. PWID have very few opportunities to come together in society free of stigma. SSPs can facilitate the development of social networks since social networks are constructed by individuals *through* interactions and institutions [73, 74].

Simmel introduced the idea of social spaces as key facets to community life in his 1949 publication, *The Sociology of Sociability*. Here Simmel discussed the importance of individuals coming together in spaces, such as churches and clubs, to socialize. Simmel found these places to be particularly important because people could be in “union with others” [75]. Later, in 1989 Oldenburg crystalized the importance of space in social relations by identifying three key spaces, home, workplace, and third places in his book, *The Great Good Place*. Oldenburg argued for society to flourish people needed to have third places to socialize that do not require a membership to enter. He said third places have eight criteria: they are *homes away from home*, *playful* by having an enjoyable atmosphere where *conversation* is the main activity, have a core group of *regulars*, are not dependent on someone’s social or economic status (i.e., in other words they are *levelers*), are *neutral ground* where people can come and go as they please, are *accessible* by walking, and are *accommodating* by being available at convenient times [76, 77].

Klinenberg further developed the concept of third places in his book which argues that libraries are key *social infrastructures* [78]. Social infrastructures are spaces where people in society come together and where communities can be formed. These can be public spaces, such as parks or libraries, or private spaces such coffee shops. Although PWID can access most of the social infrastructures mentioned, these spaces usually are not welcoming of PWID and often are exclusionary because of drug use stigma [79]. For example, public parks are policed and even when they are not, people who are homeless or use drugs often do not feel like it is a home because of the way other park goers treat them [80]. In this paper we argue that SSPs can be the social infrastructure needed to combat drug use stigma.

## Data and Methods

### Recruitment Methods

Participants were recruited from two syringe service programs (SSPs), one in in rural southern Illinois and one in New York City (NYC). The Illinois SSP had two physical locations but functioned mostly as mobile outreach by having a van that would deliver products and services to participants’ homes across the 16 southernmost counties of Illinois. The NYC SSP was a fixed location in Manhattan and as such offered various peer groups, computers, phone service, internet access, and food, in addition to other services. In Illinois, the authors partnered with an existing research project, Ending Transmission of HIV, HCV, and Overdose in Rural Communities of People Who Inject Drugs (ETHIC) who they had been working with for three years [28, 79, 81, 82]. As such, the study staff in Illinois had strong community ties as they had been working with the PWID population and the SSP. Illinois participants were identified in collaboration with study staff on the ETHIC project and contacted via telephone or in person by our partner SSP to schedule an interview for this study. The NYC SSP was identified through past networks; however, the authors had not been working at the SSP within the last three years. To build trust and rapport study staff spent one day per week at the SSP learning about the community, talking with participants and staff, and conducting ethnographic observations and interviews. The main staff person who conducted most of the interviews in NYC had a history of injection drug use, which was disclosed to participants and helped facilitate trust and rapport.

Inclusion criteria for the study was being 18 years of age or older, proficient in English, and having injected drugs at least once within the last year; however, most participants injected drugs multiple times within the last 30 days. In Illinois, participants were selected through the ETHIC study. In NYC, participants were selected in collaboration with the SSP where study staff conducted ethnographic observations weekly. Participants were screened again by the study staff who interviewed them before the interview. All participants identified who were recruited to participate met eligibility criteria and consented to participate in the study. We interviewed 57 PWID, 18 from Illinois and 39 from NYC, from August 2019 through February 2020. In February 2020, we stopped data collection in both areas due to COVID-19 concerns, thus results reflect pre-COVID-19 realities. In both areas we believe we reached theoretical saturation, meaning the interviews conducted were not producing new data nor new themes or categories in relation to PrEP when we stopped interviewing [83, 84]. Informed consent was obtained from all participants. After each interview, study staff immediately created a memo describing their observations and experiences [85]. Each participant received a $40 visa card for participating. All protocols were approved by the institutional review board at New York University and a reliance agreement was signed with Southern Illinois University for collaborations with the ETHIC project.

### Interview Structure

Semi-structured interviews were conducted in-person and audio recorded. Interview guides were informed by past research. Once they were developed, they were shared among an interdisciplinary research team as well as with people who had a history of using drugs non-medically for feedback. The final guides covered the following domains: experiences with healthcare and SSPs; HIV knowledge and prevention; PrEP care continuum stages; stigma; fentanyl awareness, knowledge and experiences, including using fentanyl test strips; overdose and naloxone; and general drug-use behaviors. General HIV knowledge was asked prior to the PrEP questions through an open-ended question asking, “Please tell me what you know and think about HIV?” After participants talked about HIV, and appropriate probing occurred if needed, they were then asked about PrEP. The first question asked was, “Have you ever heard about a pill you can take daily to prevent HIV BEFORE being exposed to HIV, this is sometimes referred to as PrEP?” If participants answered yes to hearing about PrEP, they were then asked a series of questions eliciting narratives of what, how, where, and when they heard about PrEP, including questions about PrEP costs. Participants were then read the following script “Pre-exposure prophylaxis, or PrEP, is an antiretroviral medicine, such as Truvada, taken for months or years by a person who is HIV-negative to reduce the risk of getting HIV.” If a participant answered no to hearing about PrEP, we immediately read the above script. After reading the script participants were asked if they ever tried to get PrEP and if they would be interested in taking PrEP. We then asked a series of questions about potential barriers to, and facilitators of taking PrEP, such as taking a pill daily, follow-up appointments if taking PrEP, PrEP cost, social support to take PrEP, what they think about people who are taking PrEP, and any other worries or concerns they might have in relation to PrEP [86]. Participants were also asked about their willingness to use injectable PrEP and where they felt the best place to engage in PrEP care was for people who use drugs. Demographic characteristics including age, gender, sexual orientation, education, and employment were collected at the end of each interview. Interviews ranged in length from thirty minutes to two hours, with most lasting close to two hours.

### Data Analysis

Audio files from the semi-structured interviews were reviewed and professionally transcribed. Informed by grounded theory, constant comparison and theoretical sampling methods [83, 87], the interview transcripts and memos were immediately reviewed for accuracy and assigned to one of three coders for coding. This was done after every interview so that so that adjustments to the qualitative guide and/or recruitment could be made. Constant comparative methods are used to generate hypotheses about a general phenomenon. This method is not meant to produce generalizable results or insights [87]. Qualitative methods, such as these, provide deep description and insight into contextual factors, and help describe the ways in which peoples’ lived experience may differ by setting. Qualitative comparisons of different localities have provided insights into the social construction of gender [88], intimate violence victimization [89], and HIV [90].

To ensure trustworthiness of the data we triangulated the data with ethnographic observations, qualitative interviews, memos from ethnographic observations, memos from qualitative interviews, and memos from coding [91]. Although the data are not generalizable, to address transferability, we included two research sites and purposively sampled PWID engaged at the partnering SSPs. Having three coders looking at the data assisted with ensuring dependability and confirmability. Finally, we shared our results with our community partners to ensure credibility [92, 93]. Data were processed and analyzed using Dedoose (Version 8.3.17). Relevant themes were compiled in a qualitative codebook as they emerged from the data, and the codebook continued to change throughout the coding process as new themes emerged [83, 94]. Codes were reviewed through dialogue and a final consensus was reached among the three coders. After all interviews were coded, we reviewed codes and re-coded as needed along the four PrEP care continuum stages (awareness, knowledge, willingness, and uptake). We then sub-set the data and examined by geographic location (rural versus urban) to identify potential differences and similarities along the PrEP care continuum codes. All participants have been given pseudonyms to protect their identities [95].

## Results

Thirty-nine interviews in NYC and 18 in rural southern Illinois were conducted with PWID. Demographic characteristics differed by site, particularly by race, ethnicity, and age (Table 1). However, the demographics generally reflected the overall population of each location, with NYC being more diverse in gender, race, and ethnicity. Among the 57 PWID interviewed we found that engagement in the PrEP care continuum was low, especially among the rural population, with no PWID taking PrEP. Below, we describe PrEP care continuum engagement by location. Table 2 summarizes the continuum findings.

**Table 1:** Sociodemographic characteristics of 57 people who inject drugs in rural southern Illinois and New York City, 2020

	Rural Southern Illinois n = 18	New York City n = 39
Age^a^		
18–30	6 (33%)	6 (16%)
31–40	6 (33%)	10 (26%)
41–50	5 (28%)	16 (42%)
51–60	1 (6%)	6 (16%)
Gender		
Male	10 (56%)	24 (62%)
Female	8 (44%)	13 (33%)
Transgender female	0 (0%)	2 (5%)
Race/ethnicity^a^		
White	17 (94%)	8 (21%)
Black	0 (0%)	4 (10%)
Black and Hispanic	0 (0%)	6 (15%)
Hispanic	0 (0%)	19 (49%)
Native American	1 (6%)	0 (0%)
Sexual orientation^a^		
Heterosexual	16 (89%)	33 (94%)
Bisexual	2 (11%)	2 (6%)
Education^a^		
Less than high school	3 (17%)	9 (26%)
High school/GED	6 (33%)	13 (37%)
Some college	8 (44%)	12 (34%)
Bachelor’s degree	1 (6%)	1 (3%)

^a^Missing data

**Table 2:** PrEP care continuum stage involvement by location

	New York City	Rural Southern Illinois
Awareness	Most were aware. Means of gaining awareness were: 1. Mass media campaigns 2. In-person conversations (e.g., SSP and social networks)	About half were aware. Means of gaining awareness were: 1. Mass media campaigns
Knowledge	Some had accurate knowledge. Means of gaining knowledge were: 1. In-person conversations increase knowledge	None had accurate knowledge. Explanation for no knowledge: 1. Media campaigns did not provide accurate knowledge
Willingness	About half were willing. 1. HIV and sexuality stigma were barriers to PrEP willingness	About half were willing. 1. HIV and sexuality stigma were barriers to PrEP willingness
Uptake	No participants were taking PrEP at the time of the interview (3 previously took PrEP)	No participants were taking PrEP at the time of the interview
Injectable PrEP	Most preferred injectable PrEP	Most preferred injectable PrEP

### PrEP Awareness

#### Rural Southern Illinois

In rural southern Illinois, half (9 PWID) had heard of PrEP. Most participants in Illinois gathered information about PrEP through commercials. One participant indicated looking up more information about PrEP on the internet after seeing an advertisement, but no one else had inquired about PrEP by either looking it up online or asking another person, such as a friend or healthcare provider. No participants in Illinois had discussed PrEP with a doctor or healthcare provider for educational purposes or to acquire a prescription, although many discussed conversations with healthcare providers for other needs.

Given that the main exposure to PrEP information was through advertisements, most of which targeted sexual minority men (SMM), participants interpreted the advertisements through a cultural lens that included a particular view of same-sex relationships. For example, Paul, a heterosexual 36-year-old white man said,

Yeah, I seen this [PrEP] on a commercial. I got to watch a lot of TV in prison… it's pretty, uh, pretty gay commercial.

Paul points to the fact that the advertisement was likely targeting gay men [96, 97]. Given only two of the Illinois participants reported being bisexual, and the exclusion of people using drugs, most did not recognize PrEP as an option for themselves.

#### New York City

The majority of NYC participants had heard of PrEP (30 out of 39). Similar to Illinois, participants in NYC saw PrEP on television commercials and other advertisements. However, participants saw advertisements and flyers in harm reduction settings, such as the SSP, and many talked with peers and/or in formal settings about PrEP. For example, when asked if he had heard of PrEP, Rob, a 51-year-old Hispanic man in NYC, said:

It was a commercial and the groups here [SSP]. I heard about that.

The NYC SSP offered support groups covering varying topics. NYC participants mentioned these groups as places where they were able to gather and socialize, as well as where they could obtain a free meal. Unlike participants in Illinois who mainly gathered information about PrEP from advertisements on television or radio, and had no other information source, participants from NYC talked about a combination of advertisements and conversations about PrEP, highlighting the importance of having a physical location where they could form community and exchange information. This combination increased PrEP awareness and for some began the process of developing knowledge about PrEP.

### PrEP Knowledge

#### Rural Southern Illinois

No participant in Illinois accurately describe PrEP. A common misconception was that PrEP was a drug exclusively used to treat HIV. Sheila, a 39-year-old white woman, who was informed about PrEP from the SSP, seemed to think PrEP was a pill for people who were HIV-positive. Sheila told us PrEP was,

…a pill that you can take. People with HIV, um, a pill that you can take every day, you know. But I didn't even think to ask him either because there was no cure for AIDS. So why would you, why we taking a pill?

Another participant, Alex, a 26-year-old white man, asked the interviewer, “does it help with people who have it [HIV] too?” When we explained that PrEP was for people who were HIV-negative Alex said “Ok, so no, I didn’t know how it works.”

While some participants thought PrEP was a pill to treat HIV, others understood it as prophylaxis and compared it to birth control. Most did not articulate a need for continued use before exposure to increase efficacy, which is recommended (however, the iPrEx study found that SMM had an estimated 76% protection when taking only 2 pills a week [98]). For example, Mark, a 41-year-old white man, who learned about PrEP about 2 years prior from the SSP, said,

Uh, it's like the birth control that you take the night before I guess, kind of. Uh ... I don't know.

#### New York City

NYC participants were better able to articulate what PrEP was; however, sometimes their knowledge was mixed or incomplete. Often, the inaccurate PrEP knowledge was because participants had only heard about PrEP through advertisements. Some participants conflated PrEP with post-exposure prophylaxis (PEP). For example, Lexi, a 42-year-old Hispanic woman said,

…it's a medication that helps you, it protects you from getting the HIV virus… Yeah, like a cocktail. You have to get it right away. Let's say this person shared needles with this one, and you have [to] go to your provider within I believe 42 to 72 hours in order to get that medication.

Despite some participants having inaccurate PrEP knowledge, many NYC participants articulated a clear understanding of PrEP. Better understanding was mostly among participants who received PrEP knowledge from multiple sources, including formal in-person conversations about PrEP in healthcare settings and mostly at the SSP, highlighting the importance of social infrastructures for PWID. For example, Jean, a 53-year-old Black woman who became aware of PrEP in a formal training session at the SSP said,

I heard you have to be HIV negative in order to obtain it, to get it. You take it daily and it does not prevent you from STDs and anything else. It's basically just for HIV.

### Willingness to take PrEP—Facilitators and Barriers

Willingness to take PrEP was similar in both locations. About half of the participants indicated that they would be interested in taking PrEP. The main reason for PrEP willingness was perceived HIV risk.

#### Facilitators

##### Rural Southern Illinois

Elizabeth, a 23-year-old Native American woman indicating that she believed HIV was in her social network. She said she might be interested in PrEP because,

I probably hang around a lot of people that have it [HIV].

As the interview went on Elizabeth indicated greater interest in PrEP if it would be offered at the local SSP.

Other participants focused on specific behaviors that might place them at risk for HIV. For example, Tawna, a 28-year-old white woman said she would be interested in PrEP,

Because I am an IV [intravenous drug] user, and there's always that risk. I mean, even not being an IV user there's a risk

#### New York City

Like in Illinois, NYC participants viewed PrEP as potentially viable and valuable because they were concerned that they might be exposed to HIV. For example, Cesar, a 34-year-old Hispanic man who previously was on PrEP recounts an experience where he shared injection equipment. Cesar said,

The hardest decision was not having a fucking needle knowing the guy next to you has got full blown AIDS and you got to do a bag because you’re going to fucking die.

Jason, a 48-year-old Hispanic man in NYC who had previously had HCV and was treated for it, told us he was trying to use new injection equipment. He spoke mostly about his injection risk because at the time of the interview he did not have a current sexual partner. When we asked if he would be interested in PrEP, he said,

…the way that I'm going, for a person like me, hell yeah, I would try it.

Although about half of the participants in each location expressed an interest in PrEP, half did not. Reasons for not being interested in PrEP are discussed below.

##### Barriers

Lack of perceived HIV risk was the main reason for not being interested in PrEP. When participants discussed risk, they mostly referred to sexual risk. Additionally, when participants talked about their HIV risk, they often made statements or use language that differentiated themselves from people who either have HIV or would need to protect themselves from HIV. These distinctions were stereotyping and stigmatizing [99], and operated as barriers to PrEP.

##### Rural Southern Illinois

Stigma, due to drug use, sexualities, and HIV was more pronounced in Illinois than in NYC. Rural life, as PWID in Illinois described it, included a small-town feel, poverty, and a lack of available health services for participants [79], all of which exacerbated stigma, and consequently reduced willingness to take PrEP. For example, Tawna, a 28-year-old white woman said,

Currently I’m not going for healthcare and I probably should cause I got a lot of health problems, but uh…because they look at you like you’re different, if you’re a drug addict…it’s just yet again a small town, small community.

Drug use stigma permeated almost all interactions for participants in Illinois who were concerned about community members knowing their whereabouts [81]. Participants could avoid local care by traveling elsewhere, thus mitigating stigma, but most participants did not have the means to go out of town. The rural landscape was vast and made it difficult to get places, including medical appointments. Transportation difficulties, coupled with the thought that others would know why they were going to the doctor, and that their drug use would be a source of discrimination, deterred participants from health care in general, and PrEP specifically. For example, Christina, a 38-year-old white woman, when asked if she would go to follow up doctor appointments to review if she would still benefit from PrEP said,

It depends where it is located at. I mean, because, I don’t have transportation as it is.

Stigma and rurality also prevented participants from considering PrEP because they did not perceive themselves as being one of “those people” who would need to prevent HIV and made stigmatizing comments due to HIV and sexuality. For example, when asked if he would be interested in taking PrEP, John, a 29-year-old white man said,

It's a no. I mean, I stay with the same people. I don't really go around sleeping around, I don't, I mean, I been with three women in a year, almost two years. I mean, I don't think that's that bad, you know what I mean? Especially just coming out of prison for six years straight. That's not bad, you know.

Similar to John, Melissa, a 36-year-old white woman, did not feel she was at risk. When asked if she was interested in taking PrEP, like John she references sexual risks, with no mention of injection by saying,

I mean, no because honestly, I don't sleep around like that.

HIV risk perception is not always accurate [34]. Part of this problem may reflect that most PrEP messaging has been around sexual risk, with little to no attention to drug use behaviors, including injection behaviors [65]. The other part may be because participants have actively stigmatized PrEP via HIV and sexualities stigmas. Participants used language to distance themselves from people who have HIV/AIDS, suggesting that they were different than *“those”* type of people. By doing this, participants may have convinced themselves that they were not at risk for HIV [100]. For example, Steve, a 30-year-old white man, when asked if he would be interested in PrEP, said,

Um, sure. If I ever knew that I was going to be exposed to it, but I don't know why I would have sex with those people. There's tons of people to have sex with, why would you pick somebody with HIV?

##### New York City

In contrast to Illinois, NYC participants lived in an urban setting where they did not have to navigate a rural landscape and small-town social relations. Participants accessed services at a fixed-location SSP that also provided a sanctuary from drug use stigma. Getting to and from the SSP, along with other local services, was easy to do by taking accessible and low cost public transportation and/or walking [62]. These structural differences also impacted stigma. Although HIV stigma emerged as a barrier to willingness to take PrEP in NYC, it was not as pronounced as in Illinois. In addition, many participants knew, and cared about, people who were HIV-positive, which combatted HIV stigma. For example, Calie, a 30-year-old Black and Hispanic woman told us she would be interested in taking PrEP because her “father passed away from HIV” and her “roommate is actually HIV-positive.”

It is not surprising that NYC PWID mentioned knowing people living with HIV. NYC was the “epicenter” for the HIV/AIDS outbreak in the 1980’s and had the largest share of new HIV infections in the United States (34% in 1985) [101]. PWID were heavily impacted by HIV/AIDS in NYC with about 50% being HIV-positive in 1984–1987 [102]. Thus, there is a historical and perhaps even collective memory [103] of HIV in NYC, one tied to injection drug use and same sex behavior, and many PWID knew people who had died.

Memories of HIV often sparked fear among participants. For example, when we first asked about HIV, Calie said, “That [HIV] I don’t have, thank God.” Similarly, Rob, a 51-year-old Hispanic man said “I don’t have that [HIV] either. Thank God.”

Responses about HIV contrasted heavily to responses to other diseases, such as hepatitis C virus (HCV), another chronic illness common among PWID. This may be because participants, given their experiences and memories about HIV, did not view HIV as chronic, but rather as deadly. For example, Cesar, a 34-year-old Hispanic man, talked about having HCV a few times and clearing it each time. Although he said he hoped to not get HCV again, he did not think of HCV as a terrible death, like he thought of HIV. Cesar said:

I’m going to be honest with you. Most teenagers I know have Hep-C. Most men my age in this neighborhood have fucking full blown AIDS, not even HIV at this point. 34. 34 years old dude.

Fear of HIV may have propelled Cesar to use PrEP because he told us that he had previously been on PrEP, but that he stopped using it because he did not go back to refill his monthly prescription. When we asked him if he would be willing to take PrEP in the future he said, “If I need it, I’ll go get it.”

Josh, a 47-year-old white man, who was not interested in PrEP because he did not feel he was at risk, told us that he wanted to kill himself when he thought he might have tested positive for HIV. Josh did not talk about knowing people with HIV and seemed to harbor more HIV stigma than Cesar. Josh said,

When I first come home from prison in 2009, I was trying to get back with this lady I was with. So, I run, and get all the tests. So, I'm sitting in the doctor's office the first time I ever had the AIDS test, and she's like, "Oh, I'll be back in 20 minutes." Two hours go by, I start thinking to myself, "Oh, my God. I got it. I'm gonna go to the roof, I'm gonna jump off the roof."

Although not as prominent as in rural Illinois, some NYC participants talked about HIV in stigmatizing ways even though they knew HIV-positive people. The stigma was mainly in relation to sexualities and echoes the advertisements that PrEP is for sexual minority men. For example, Joey, a 45-year-old Hispanic man in NYC said,

A few months ago, my boy had a surgery. He had thyroid cancer. He is actually the one that had HIV. And it was a conversation. Health-wise, as far as he was concerned. A very good friend of mine so we got into a long conversation about how he was not taking care of himself. He was not taking his meds, and he was doing drugs, and da-da. And then that came up. But he would not get back on meds, he kept going in the hospital [hospital name retracted] and they offered, actually, they actually thought that me and him were partners, and they offered me the drug. And I was like ‘this is my damn cousin, let me alone. I am not gay. I am not bisexual. I do not put myself in that position.’

Joey had first learned about PrEP when a doctor tried to talk to him because, as Joey says, the doctor thought he was in a sero-discordant sexual relationship (i.e., a relationship where one person is HIV-positive, and the other is not). Yet, Joey clearly states that he would not be in a relationship like that by demarcating sexual minorities as other [99].

### PrEP Uptake

There were no participants who were on PrEP at the time of the interview in either location. However, three NYC participants had previously taken PrEP. Given the lack of PrEP uptake in our sample, we asked hypothetical questions to participants to gauge where they would like to receive PrEP care and what potential barriers to PrEP might occur. The overwhelming response that we received was that participants preferred to receive PrEP at their local SSP because of the friendly environment and trust they had at the SSP [62]. For example, Tawna in Illinois said she would prefer PrEP at her SSP “cause that would cut out doctors.” Similarly, in NYC, Rick, a 51-year-old Hispanic man told us that PrEP would be best “in drug places like this, like, harm reduction.”

Finally, we asked a hypothetical question about injectable PrEP by asking “If a once-a-month shot was available as PrEP would you be interested?” When presented with this option most participants indicated injectable PrEP as easier. For example, Sheila in Illinois said that she would be

A lot more interested than taking a pill every day... because it's just easier, I don't have to carry, like right now I don't have a purse on me... I don't have to worry about them getting stolen or whatever. And- or explaining to people, you know, hey, what it is or whatever.?

Participants felt getting a shot once a month was much easier than taking a pill a day and would afford more privacy. However, it should be noted that most participants indicated that they would not have trouble taking oral PrEP daily.

## Discussion

This study sought to identify barriers and facilitators to PrEP care continuum engagement in rural Illinois and urban NYC. More participants in NYC were PrEP aware and better explained what PrEP was than participants in rural Illinois. We identify the fixed location SSP in NYC where PWID established community and social ties as a facilitator of PrEP. In rural southern Illinois, where the SSP was mobile, no participants accurately described PrEP; thus, none of those participants made it to the second stage of the PrEP care continuum (i.e., PrEP knowledge). After we described PrEP to participants, we found that about half of the participants in both locations were interested in taking PrEP. A significant barrier to PrEP continuum engagement was stigma due to drug use, HIV, and sexualities.

Past research on PWID has shown lack of PrEP awareness [5, 8, 65, 104–106] and lack of accurate PrEP knowledge as barriers to PrEP uptake [105]. Yet, once aware and knowledgeable about PrEP, PWID are interested in taking it [5, 107]. Similar to other studies, participants in this study described perceived low HIV risk as a reason for not being interested in taking PrEP [108]. Although participants may have accurately described their risk, past research has shown that perceived HIV risk does not always match actual risk [34] and a recent study found that 92% of PWID sampled in Massachusetts met CDC indications for PrEP [109]. One reason that PWID might miscalculate their risk is because they think about HIV-risk in terms of sexual risk, particularly “gay” sexual risk [110]. Thus, stigma due to sexualities may have played a part in willingness to take PrEP.

In addition to stigma due to sexualities, stigma due to drug use and HIV operated as barriers to willingness to take PrEP. Stigma was more pronounced in the rural setting due to the small town sociability patterns where everyone knew each other’s activities and whereabouts and where participants faced discrimination from community members, healthcare providers, and law enforcement [79, 81]. In NYC, collective memories and trauma [103, 111] associated with HIV evoked a sense of fear among participants, but at the same time these memories humanized people with HIV, thus mitigating HIV stigma.

One way to combat PrEP stigma is by creating sex and drug positive messaging [112, 113]. PWID face stigma in their exclusion from PrEP marketing, which can translate into lack of PrEP awareness and knowledge, and thus halt engagement in subsequent care continuum stages. In 2014, the NYC Department of Health and Mental Hygiene (NYC DOHMH) launched inclusive PrEP campaigns [114] but they focused mainly on sexual transmission. Although these campaigns may have contributed to greater PrEP awareness amongst NYC participants, the exclusion of drug use messaging likely operated as a barrier to PrEP.

Another stigma reduction tool that would support PrEP care engagement could be packaging PrEP in important social infrastructures, such as SSPs. Research has identified SSPs as important for disseminating PrEP awareness [64, 65, 115]. Indeed, NYC participants reported learning about PrEP at their SSP and had greater PrEP knowledge because of conversations at the SSP. The atmosphere of the SSP’s fixed location in NYC was informal, allowing participants, many of whom were homeless, to have a space to congregate and be comfortable. Participants used the SSP as a refuge from weather and a safe place to talk with others like themselves, and thus avoided stigma outside the SSP. This atmosphere facilitated conversations, and in particular information exchanges about PrEP. Overall, the SSP was a key space where social networks developed and were fostered.

Although this study highlights the importance of physical spaces for social networks to be cultivated, mobile SSPs can still spread PrEP awareness and knowledge. In the absence of a physical location, mobile SSPs could partner with community members to activate social networks to increase care continuum engagement [116]. Another possibility could be designating spaces that a mobile unit goes to at scheduled times so that community members can foster relationships. Mobile SSPs could also consider having a PrEP prescriber on the mobile unit while fixed-locations can offer care on site [117, 118]. In conjunction with the above recommendations, it may be beneficial to consider same-day PrEP prescribing and medication provision [119, 120]. Given that PWID may have competing priorities, including basic needs (e.g., food, housing, employment, etc.), limiting the number of visits required for PrEP initiation and maintenance could significantly reduce PrEP care continuum drop off [121]. Importantly, PrEP initiation is only one step in the care continuum and later steps such as persistence and retention will need support [86]. Finally, long-acting injectable PrEP may be of particular importance. Participants noted the ease of injectable PrEP as it would not require a daily pill and would be more discreet.

There were limitations to this study. First, participants were SSP clients. Thus, the experiences of these individuals may not generalize to all PWID. Second, the International Network of People Who Use Drugs (INPUD) has raised concerns about PrEP that did not emerge in this study such as concerns about the effectiveness of PrEP in preventing HIV transmission via injection as well as concerns about resource distribution of antiretroviral therapy (ART) and the political support for allowing SSPs to exist and fund the provision of new injecting equipment if PrEP is prioritized. Future studies should explore these concerns. Third, the findings may not be generalizable to all PWID as treatment landscapes, neighborhood conditions, and structural factors likely differ by area. Fourth, this study was not able to explore PrEP continuation after PrEP uptake since no participants were currently on PrEP. Fifth, the sample sizes differ by location, however, theoretical saturation seemed to occur in both locations. Finally, interviews were conducted prior to the COVID-19 pandemic. The recent pandemic may exacerbate HIV risk among PWID [122], and therefore, PrEP may be especially important now.

In conclusion, we suggest tackling stigma as a fundamental cause of PrEP inequalities, and more generally of poor health outcomes, among PWID [44, 59]. We strongly suggest partnering with local SSPs for delivering PrEP care. PrEP can be packaged as one of the many harm reduction tools offered, including drug treatment and medications for opioid use disorder. Creating social infrastructures for PWID is a critical facet to de-stigmatizing drug use and supporting the diffusion of PrEP. Thus, funding for SSPs to remain open and provide valuable resources, including PrEP, is important [123].

## Supplementary Information

Below is the link to the electronic supplementary material.

Supplementary file1 (DOCX 24 kb)
