# Supplementary material for: PrEP Care Continuum Engagement Among Persons Who Inject Drugs: Rural and Urban Differences in Stigma and Social Infrastructure
Source: AIDS Behav. 2021 Oct 9;26(4):1308–20. doi: 10.1007/s10461-021-03488-2 (PMC8501360; doi:10.1007/s10461-021-03488-2)
Supplement: Supplementary file 1 — Supplementary file1 (DOCX 24 kb) [file 10461_2021_3488_MOESM1_ESM.docx]

**Interview Guide for PWID**

To be eligible they need to have injected drugs within the last 30 days:

The above will be pre-determined by selecting [community partners - redacted for anonymity].

Interviewer please go through the consent process. Make sure to give the incentive at the time you do consent.

Begin recording and say the below script:

“Today is [date]. This is an interview with [interview code number]. Before we start the interview, I'd like to say that I'm recording you, and ask if you give me your permission to record you.”

**Introduction**

Interviewer script: Thank you for talking to me today. We want to improve health services for people who use drugs in [insert area] and in order to do that we would like to know a little about your life, your opinions, and your experiences. If you are uncomfortable with any questions, and do not want to answer them, please let me know and we can skip them.

We are really interested in discussing what you know and think about these issues. I would like to emphasize that there are no right or wrong answers to any question.

Everything you tell me will be kept confidential and we will not share your name with anyone besides study staff.

Do you have any questions before we start? (Take time to address all questions and concerns)

**Note to interviewer: please turn off your cell phone and other mobile devices.**

Interviewer Script: I am going to start by asking questions to get to know you and [SEP name] a little better

**SEP**

1. Can you tell me a little about your experiences with [insert SEP name]?
2. What do you like most about [insert SEP name]?
3. What do you like least about [insert SEP name]?
4. Please tell me about a time you wanted to come to [SEP name] but did not.
   1. What were the reasons you did not come?
   2. SEP Probes: How do you get here? How often do you come here? What happens when you come here? (probe: what kind of services do you get?) **OR** tell me what a typical visit is like.
5. Can you tell me about other places you get syringes and equipment for injecting?
   1. Can you please walk me through a week and explain how this works? If you get syringes from multiple sources, please let me know.
      1. Probes: how close is [location they get syringes] to where they live? How easy is it to get there and obtain syringes? Do they have to pay for syringes?
6. Can you tell me about a time you were not able to use new injecting equipment?
   1. Probe: How often are you able to use new and sterile syringes and injecting equipment such as cookers, water, and filters?

Interviewer Script: Now I am going to ask some questions about your experiences with health care. I appreciate you sharing your expertise on these areas with me. If you have any questions or need a break, please let me know.

**Healthcare/SEP**

1. Where are you most comfortable going for health services?
   1. Why? Please explain what your experiences are.
2. What places do you hate to go to for health services?
   1. Why? Please explain what your experiences are.
3. Tell me about your most recent interactions with any doctor or health care provider?
   1. How did you get there?
   2. Did you discuss your drug use? (why/why not)?
4. Please tell me what you think about the Department of Health in [insert location].

Interviewer Prompt: I am now going to ask questions about HIV and hepatitis C (HCV). If you have any questions or need anything please let me know.

**HCV**

1. Please tell me what you know and think about HCV?
2. Is HCV common among the people who hang out with?
3. How is HCV transmitted?
4. Who do you think is most at risk for HCV?
5. Have you or someone you know been treated for HCV?
   1. Can you describe what the treatment is like? (ask when they were treated if they can do this)
   2. Were there any side effects?
   3. Can you describe what the most current HCV treatment is like?

**HIV**

1. Please tell me what you know and think about HIV?
2. Who do you think is most at risk for HIV?
3. Have you ever heard about a pill you can take daily to prevent HIV BEFORE being exposed to HIV, this is sometimes referred to as PrEP?

If yes, ask the following questions, otherwise skip to the interviewer script and tell them what PrEP is

- 1. What have you heard?
  2. Where did you hear about PrEP?
  3. When did you hear about PrEP? Please describe how you heard about PrEP so I can have a full picture of what happened.
  4. Can you tell me how to get PrEP?
  5. Can you tell me how much PrEP costs?

Interviewer Script: PRE-exposure prophylaxis, or PrEP, is an antiretroviral medicine, such as Truvada, taken for months or years by a person who is HIV-negative to reduce the risk of getting HIV.

1. Who do you think would benefit most from PrEP?
2. Have you ever tried to get PrEP?
   1. If yes, say “Please tell me the process of trying to get PrEP? What was that like? Did you get PrEP? Are you on PrEP? How long did you stay on PrEP? If off PrEP ask, Why did you get off PrEP?
   2. If no, ask “Why not?”
3. Would you be interested in taking PrEP to prevent HIV?
   1. Why/Why not?

Interviewer Script: The next few questions I am going to ask you are about PrEP as well. Please answer them to the best of your ability.

1. In order to take PrEP, you’d have to take a pill every day. Do you think you could take PrEP every day? Why/why not?
2. Please tell me about your experiences taking a pill, drug, or anything else daily, if you have any.
   1. Please describe the process.
3. If a once a month shot was available as PrEP would you be interested? Why? Why not?
   1. Probe to see if they prefer a once a month shot or a pill a day. Do they think others would prefer this?
4. In order to take PrEP, you’d have to go to the doctor every 3-6 months in order to get tested for HIV and review if you would still benefit from PrEP. Is that possible for you?
   1. Why/why not?
5. How much would you be willing to pay for PrEP each month to prevent HIV infection?
   1. Probe: Why? Would you take it if free?
   2. If unwilling to pay or uninterested in PrEP for themselves ask the following: “How much do you think a friend would be willing to pay?”
6. Would your partner, if you have one, support you taking PrEP?
   1. Why/why not?
   2. If they are uninterested in PrEP ask a hypothetical: “Do you think partners of people you know who could benefit from PrEP would encourage them to take PREP? Why/Why not?”
7. Do you have any worries or concerns about PrEP? Please tell me about them.
8. Where do you think the best place to offer PrEP for people who use drugs is? Why?
9. Would you be interested in taking PrEP if it were offered at [insert SEP name] and your follow up appointments would be at [insert SEP name]?
   1. Why/why not
   2. If they are not, ask if they think others would be.
10. What would you think of your friends if they were taking PrEP?
    1. Please explain why.

Interviewer Script: Now I am going to ask you some questions about your drug use. I would like to restate that everything you say is confidential. Some of these questions may make you uncomfortable and if you feel you cannot answer them, we can skip them. Please try to be as accurate as possible when answering these questions. I am can talk through them if you need and before we start, I want to thank you for sharing your experiences with me.

**Stigma**

1. Have you ever been treated differently because you used drugs? If so, please tell me what happened.
   1. Probes: Has a family member treated you different? A healthcare worker? Syringe exchange? Substance use treatment? Police?
   2. Note: for each example ask the following: “Has this changed your relationship with XX?”

**Fentanyl**

1. Tell me about your experiences with fentanyl?
2. Tell me what you have heard about fentanyl strips?
3. Tell me about your experiences using fentanyl strips, if you have any.
   1. Probes: Where did you obtain the fentanyl test strip?
   2. What drugs have you used the fentanyl test strip on?
   3. How often do you use fentanyl test strip?
      1. Are there times when you will not use them? Why?
   4. Can you walk me through how you use fentanyl test strips?
   5. What do you do if your drugs test positive for fentanyl?
   6. Do you plan on using test strips in the future? Why/why not?

**Narcan/Overdose**

1. Now I’ll ask about your experience with overdosing, by overdose I mean passed out, turned blue, or stopped breathing from using drugs. Have you ever seen a person overdose?
   1. If yes, say “please tell me what happened.”
2. Have you ever overdosed?
   1. If yes, say “Can you please tell me what happened?”
3. Have you heard of Narcan or Naloxone, which can reverse an overdose? If yes, please tell me what you think about it.
   1. If yes, ask: Are you trained to recognize and respond to an overdose with Narcan or Naloxone? Why/Why not?
   2. Have you ever administered Narcan or Naloxone?
   3. Would you be comfortable carrying Narcan/Naloxone? Why/ Why not?
   4. Probe: When and where are they comfortable? Uncomfortable? Why?

**Pain**

1. Please tell me about any time in your life where pain was a problem for you?
   1. What happened? Please tell me about it.
      1. Probe for physical and mental/emotional pain

**Criminal Justice**

1. Please describe any interactions you have had with police?
2. Have you ever been arrested?
   1. How many times?
   2. Please describe
3. Have you ever been incarcerated?
   1. Please describe

**Drug Use**

1. Tell me about the types of drugs you use?
   1. When do you use drugs?
   2. How do you use drugs?
   3. Who do you use drugs with?
      1. Probe by type of drug
2. How do you get drugs?
   1. Probe by type of drug; what is the drug, set and setting? Ask about their friends (identifying and understanding social networks)
   2. Do you have a home delivery system? Do they text their dealers? Are drugs delivered to them?
3. Please describe what you do on a typical day.
   1. Probe: when you are working/not working?

**Interviewer REMINDER**: Offer the participant a break if they need one.

**Demographics**

1. How old are you?
2. How do you describe your gender?
3. What is your sexual orientation?
4. How would you describe your race/ethnicity?
5. How much school did you finish?
6. Please describe your current living situation.
7. How do you support yourself, financially?
   1. Probe: What do you do for work? How long have you been doing this? What other ways do you support yourself?

Interviewer Script: We’ve talked about a lot of things today, is there anything else you would like to add? [pause and wait for an answer]

Thank you so much for taking the time to talk with me. I really appreciate you sharing your experiences. I want to give you a chance to ask me any questions. Is there anything I can answer for you?
